# Impact of Point-of-Care Ultrasound in Medical Decision Making: Informing the Development of an Internal Medicine Global Health POCUS Curriculum

**DOI:** 10.24908/pocus.v7i1.15620

**Published:** 2022-04-21

**Authors:** Michelle Fleshner, Steve Fox, Thomas Robertson, Ayako Wendy Fujita, Divya Bhamidipati, Thuy Bui

**Affiliations:** 1 Division of Hospital Medicine, University of Colorado Anschutz Medical Center CO, Aurora; 2 Cleveland Clinic, Respiratory Institute Cleveland, OH; 3 Internal Medicine, Allegheny General Hospital Pittsburgh, PA; 4 Division of Infectious Diseases, Emory University Atlanta, GA; 5 Medical Center, Internal Medicine, University of Pittsburgh Pittsburgh, PA

**Keywords:** Point-of-care Ultrasound, Global Health, Medical Education, Internal Medicine

## Abstract

Background: Point-of-care Ultrasound (POCUS) is particularly useful in low-middle income countries (LMICs) where advanced imaging modalities and diagnostics are often unavailable. However, its use among Internal Medicine (IM) practitioners is limited and without standard curricula. This study describes POCUS scans performed by U.S. IM residents rotating in LMICs to provide recommendations for curriculum development. Methods: IM residents within a global health track performed clinically-indicated POCUS scans at two sites. They logged their interpretations and whether or not the scan changed diagnosis or management. Scans were quality-assured by POCUS experts in the US to validate results. Using the criteria of prevalence, ease of learning, and impact, a framework was developed for a POCUS curriculum for IM practitioners within LMICs. Results: A total of 256 studies were included in analysis. 237 (92.5%) answered the clinical question, 107 (41.8%) changed the diagnosis, and 106 (41.4%) changed management. The most frequently used applications were the Focused Assessment for Sonography for HIV associated TB (FASH) exam, finding fluid (pericardial effusion, pleural effusion, ascites), qualitative assessment of left ventricular function, and assessment for A-lines/B-lines/consolidation. The following scans met ease of learning criteria: FASH-basic, assessment of LV function, A-lines vs. B-lines, and finding fluid. Finding fluid and assessment of LV function changed diagnosis and management most frequently, greater than 50% of the time for each category. Discussion/Conclusion: We recommend the following applications as highest yield for inclusion in a POCUS curriculum for IM practitioners within LMICs: finding fluid (pericardial effusion, pleural effusion, ascites) and assessment of gross LV function.

## Background

Point-of-care ultrasound (POCUS) is increasingly used as a powerful diagnostic tool for bedside assessment and procedures 1. Unlike complete ultrasound (US) examinations performed by technicians and interpreted by radiologists, POCUS is performed by the clinician at the bedside to answer focused, clinical questions and integrate findings into decision making and management 1, 2. With brief training, ultrasound practitioners can rapidly diagnose and treat 3. Particularly in low and middle income countries (LMICs), POCUS is more readily available and accessible than other imaging modalities 4, 5, 6. While the use of POCUS has been well-established in Emergency Medicine, there is growing recognition of its value among other medical fields, including Internal Medicine (IM) 7, 8.

POCUS has a variety of clinical applications. Lung ultrasound has been shown to be more accurate than chest radiography for consolidation, pleural effusion, and pneumothorax 9, 10. Focused cardiac ultrasound can improve qualitative bedside assessment of left ventricular (LV) systolic function, volume responsiveness 11, 12, 13, 14, 15, 16, chamber enlargement and pericardial effusion 17, 18, 19, 20, 21. POCUS can also improve diagnosis of extrapulmonary TB using the Focused Assessment with Sonography for HIV-associated TB (FASH) 2, 22. The FASH exam identifies potential ultrasound findings in six abdominal locations that may be indicative of extrapulmonary TB (EPTB) in patients with HIV coinfection and is most sensitive for those with a CD4 count less than 100. Prior studies suggest that specifically in LMICs, POCUS may change clinical management in greater than 60% of cases 3, 6, 23, 24, 25, 26. These smaller studies depict some of the novel uses of POCUS in LMICs, but there is still limited research on the highest-yield applications of POCUS by IM physicians in LMICs. 

No standardized POCUS curriculum within IM in LMICs has been established, as clinical applications are still being studied and can be region and resource specific 6. Other studies aimed at teaching POCUS in LMICs have taught various US applications and measured trainees’ competencies pre- and post-training 27; however, to our knowledge, our study is the first to collect data on which US applications are highest yield to teach and include in an IM-based curriculum in LMICs. In considering applications to include in a POCUS curriculum, a few different criteria have been proposed. Two studies used the following three criteria: prevalence, impact, and difficulty 2, 28. The Canadian Internal Medicine Ultrasound (CIMUS) group published consensus-based recommendations for an IM POCUS curriculum that agreed upon four principles: 1) applications should be selected based on clinical and/or education needs; 2) applications should be educationally feasible (cognitive and technical components); 3) content should have clinical and/or educational evidence to support its use; and 4) any unintended consequences should pose minimal risks to patients 8. Finally, a Netherlands review describes a curriculum with applications that are easy to learn, rapid to perform, frequently encountered, and preferably have a dichotomous yes/no question. Utilizing this literature, we have chosen the following criteria to model our curriculum: prevalence, ease of learning, and impact on diagnosis and management. 

We describe the highest impact POCUS applications by investigating the ability of POCUS to answer a clinical question, assist with diagnosis, and change management when used by U.S. IM residents in two LMICs. Using these results, we quantified the prevalence, impact, and ease of learning from our study and prior literature to guide curriculum development. Furthermore, we implemented a quality assurance (QA) program to validate the use of POCUS in these settings. 

## Methods

This was a descriptive study to assess the frequency and clinical utility of various POCUS applications by IM residents in LMICs. The study was conducted by residents in the Internal Medicine/Global Health track at the University of Pittsburgh Medical Center (UPMC). 

### Prior POCUS Training

At UPMC, Pulmonary and Critical Care faculty provide POCUS training to IM residents in the Global Health track. This includes a 20-hour didactic and hands-on training in image acquisition and interpretation, including education on cardiac, lung, abdominal, and lower extremity deep vein thrombosis (DVT) assessment. Training also includes instruction on logging images and the Quality Assurance (QA) system.

### Data Collection

POCUS scans were performed in two different clinical settings: Kamuzu Central Hospital (KCH) in Lilongwe, Malawi, and Georgetown Public Hospital Corporation (GPHC) in Georgetown, Guyana. These are two international clinical sites for IM residents training at UPMC. KCH is a very low-resource environment with limited access to radiography and formal ultrasound with substantial delay; it does not have a functional CT scan. GPHC has access to radiology-performed ultrasound and radiography and CT scan in some cases. Residents performed clinically-indicated POCUS scans at their respective clinical sites. Each scan was labeled with a unique, non-protected health information (PHI) identifier. Residents documented their interpretation in Google Sheets as outlined in Table 1. The images were uploaded to Google Drive and were remotely evaluated for QA by a POCUS expert in the United States within one week. This QA process is described in detail separately 29. 

**Table 1:** Log and QA Spreadsheet. Residents completed this spreadsheet for each POCUS scan that was performed and uploaded corresponding images. QA faculty completed their component of the spreadsheet for images requesting review.

**User**	**Data Entries**
Image Uploader (GH resident)	• Unique Study ID • Type of Study (Abdominal, Cardiopulmonary, Vascular, MSK/Soft Tissue) • Country • Brief description of patient’s problem • Primary Clinical Question • POCUS findings • Did POCUS answer your clinical question? (Yes, No) • Did POCUS change diagnosis? (Yes, No) • Did POCUS change management? (Yes, No) • Category (For Urgent QA, For non-urgent QA, No additional QA needed, poor quality images (do not QA), Educational Scan)

### Ethics

Approval was obtained from the University of Pittsburgh Medical Center Institutional Review Board with educational exemption, IRB #PRO18040339. This project evaluated an initiative that was already being implemented for educational purposes. This was not human subjects research, as we were studying diagnostic reasoning rather than patients or human subjects themselves. Approval was obtained from leadership at international partner sites.

### Data Analysis

**Figure 1:**
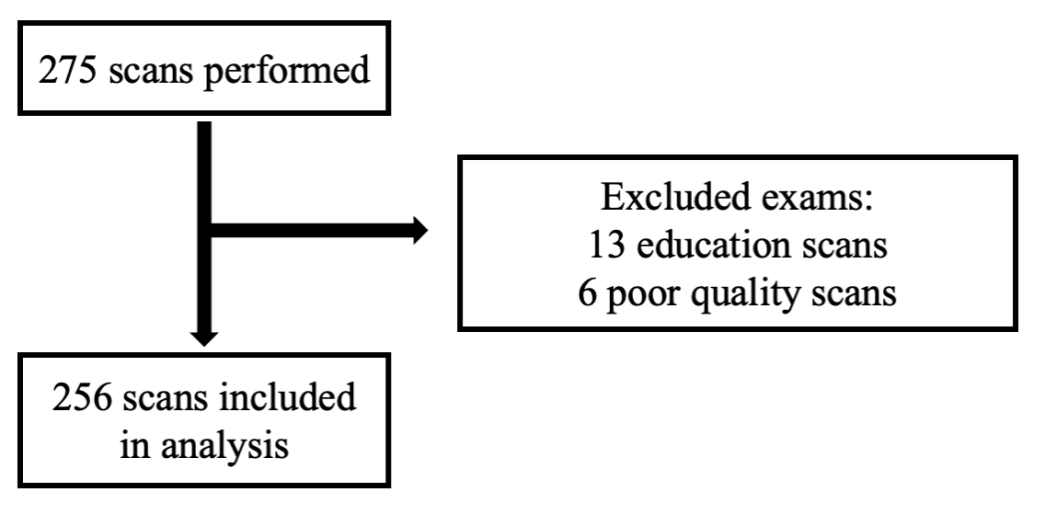
A total of 256 scans were included in the final analysis after exclusion of educational scans and poor quality scans. Educational scans were those that were only used for practice or to view a finding that was already known and not used for clinical decision making. Poor quality scans were deemed unable to be used for interpretation of any kind.

Data were analyzed using Stata/IC version 15.1 and Microsoft Excel. Studies were excluded from the analysis if they were labeled as an “Educational scan” or “Poor quality images” (Figure 1). An educational scan was a scan performed for academic purposes only and not for clinical decision making. Outcomes measured included the total number of studies performed and the number and percentage of studies that answered the clinical question, changed the diagnosis, and changed management. This was further stratified by type of study performed, location, and clinical question. Based on a prior pilot and existing literature^30^ it was felt that applications involving “finding fluid”, including assessment for ascites, pleural effusion, and pericardial effusion, may be the highest yield. Given similar technique and potential for procedural application, these applications were grouped together in analysis. Finally, study validity was assessed by measuring the number of studies that underwent QA and the frequency of concordance between the reviewer and resident interpretations.

### Defining prevalence, ease of learning, and impact on diagnosis and management 

We defined the most prevalent applications of our study as those that were performed >10 times or >5% of all scans performed by all residents. To assess the ease of learning for a particular POCUS application, we sought to answer the following question: “Can providers learn and perform this application reliably in a limited time period?” We considered a “limited” time period to be a few hours of training per application, followed by 10-25 supervised clinical exams. A literature review was performed to answer the questions of prevalence and ease of learning, as outlined further in the results section. After narrowing down the POCUS applications based on prevalence and ease of learning, we utilized the results of our study to assess the impact of each POCUS application. Diagnosis and management change are frequently studied measures of the utility of POCUS in the clinical setting 3, 23, 31, 32, thus these parameters were used to measure the impact of each POCUS application. For each exam, the examiner directly answered the questions “Did this exam change the diagnosis?” and “Did this exam change management?”. For each application, percent of “yes” answers was calculated for each question to quantify change in diagnosis and management. 

## Results

**Figure 2:**
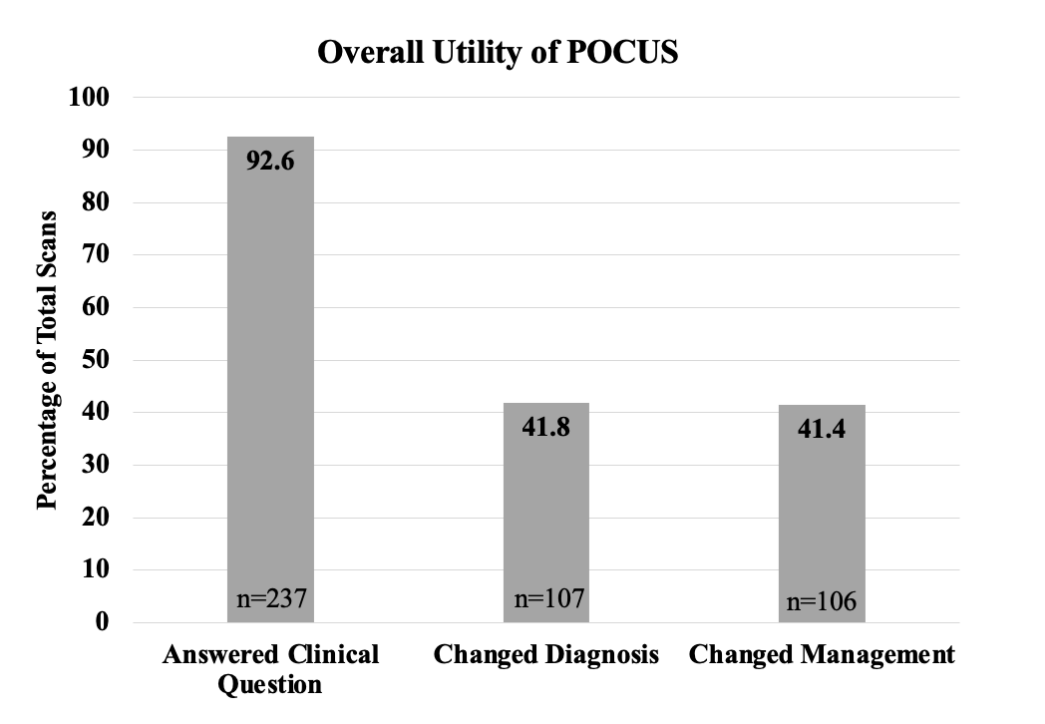
Percentage of POCUS scans that answered the clinical question, changed diagnosis, and changed management, out of 256 total scans. This was collected by survey that asked for subjective report of the individual performing the scan.

**Figure 3:**
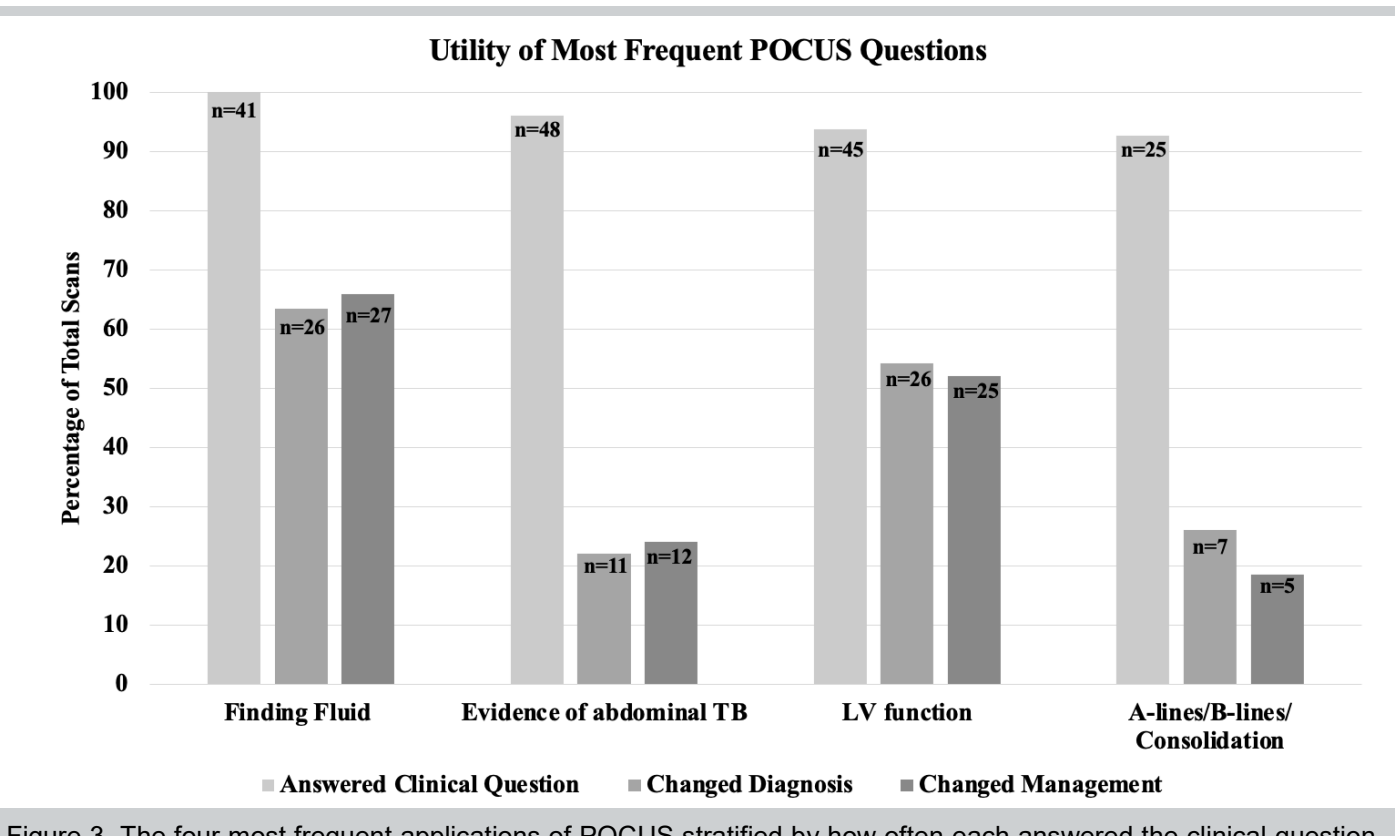
The four most frequent applications of POCUS stratified by how often each answered the clinical question, changed the diagnosis, and changed management, as subjectively reported by the individual performing the scan. The number of scans in each category is noted on top of each bar.

A total of 256 studies were included in the analysis (Table 2). 225 (88%) studies were performed in Malawi and 31 (12%) studies were performed in Guyana. The most frequent study type was cardiopulmonary with 126 (50%) studies followed by abdominal with 117 (46%) studies. Of all studies included in the analysis, 237 (92.5%) answered the clinical question, 107 (41.8%) changed the diagnosis, and 106 (41.4%) changed management (Figure 2). The majority of clinical questions were reliably answered by POCUS, however POCUS was less frequently able to answer clinical questions pertaining to: evaluation for malignancy (55.6%), assessment of RV function (77.8%), etiology of undifferentiated abdominal pain (66.7%), and assessment for vegetations (33.3%). Of the four most frequently asked questions, qualitative assessment of LV function and finding fluid changed the diagnosis and management more often than assessment for TB and A-lines/B-lines/consolidation (Figure 3). Other notable clinical questions for which POCUS frequently changed the diagnosis and management were evaluation for kidney size/ assessment of chronic kidney disease (CKD), and assessment of bladder or Foley catheter, though these were performed less frequently. All clinical questions and their ability to answer the clinical question, change diagnosis, and change management can be seen in Table 3. 

**Table 2:** Total number of studies stratified by location and type of study.

**Exams performed**	**n (%)**
Total	256 (100)
Malawi	225 (88)
Guyana	31 (12)
**Type of Study**	
Abdominal	117 (46)
Cardiopulmonary	126 (50)
Vascular	8 (3)
MSK / Soft tissue	3 (1)

**Table 3:** Clinical questions in order of frequency, broken down by how often POCUS was able to effectively answer the question, how often POCUS changed the diagnosis, and how often POCUS changed management. Items excluded from Table 4 were: “Other” and those with <3 scans which included gallbladder pathology, abscess and lung sliding.

	**Total**	**Answered Clinical Question Answered**	**Changed** **Diagnosis**	**Changed** **Management**
	**n**	**n (%)**	**n (%)**	**n (%)**
Total	256	237 (92.6)	107 (41.8)	106 (41.4)
Is there evidence of abdominal TB?	50	48 (96)	11 (22)	12 (24)
What is the qualitative LV function?	48	45 (93.8)	26 (54.2)	25 (52.1)
Finding Fluid Pleural effusion Pericardial effusion Ascites Abdominal free fluid (i.e.FAST)	41 24 7 3 7	41 (100) 24 (100) 7 (100) 3 (100) 7 (100)	26 (63.4) 19 (79) 2 (38.6) 2 (66.7) 3 (42.9)	27 (65.9) 20 (83.3) 2 (28.6) 3 (100) 2 (28.6)
Are there a-lines, b-lines or consolidation?	27	25 (92.6)	7 (26)	5 (18.5)
Is there evidence of cirrhosis?	16	15 (93.8)	7 (43.8)	5 (31.3)
Evaluation for malignancy	9	5 (55.6)	5 (55.6)	4 (44.4)
Is there evidence of DVT?	9	9 (100)	4 (44.4)	5 (55.6)
Is there hepatosplenomegaly?	9	9 (100)	2 (22.2)	1 (11.1)
Is there right ventricular (RV) strain?	9	7 (77.8)	4 (44.4)	4 (44.4)
Is there evidence of CKD? (or assessment of kidney size)	8	8 (100)	5 (62.5)	5 (62.5)
What is the volume status?	6	6 (100)	1 (16.7)	4 (66.7)
Assessment of bladder or foley	4	4 (100)	3 (75)	3 (75)
Is there hydronephrosis?	4	4 (100)	1 (25)	1 (25)
What is the etiology of abdominal pain?	3	2 (66.7)	2 (66.7)	1 (33.3)
Are there vegetations?	3	1 (33.3)	1 (33.3)	1 (33.3)

### Prevalence

**Figure 4:**
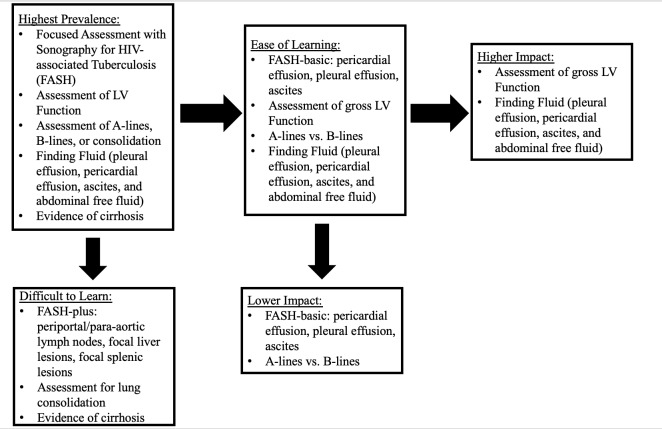
Proposed Curriculum for POCUS Education of Internal Medicine Curricula in Resource-limited Settings using Prevalence, Ease of Learning, and Impact as criteria for inclusion.

The most prevalent applications in our study were the FASH study for abdominal TB, qualitative assessment of LV function, finding fluid (included ascites or abdominal free fluid, pleural effusion, or pericardial effusion), assessment for A-lines/B-lines/consolidation, and evidence of cirrhosis. This is in relative agreement with other studies from LMICs^33^, ^34^, ^35^ with the exception of OB/GYN ultrasound being one of the most common applications in each of these studies. Both of our clinical sites had separate OB/GYN departments that performed ultrasound exams which likely explains this discrepancy. In addition, according to the CDC, HIV/AIDS and TB are among the top ten causes of mortality in Malawi 36, which supports the high use of the FASH exam in our study population. Figure 4 outlines our process for developing a POCUS curriculum, starting with the applications from our study that we have included as the “highest prevalence.” 

### Ease of Learning 

As far as ease of learning, our literature review revealed data behind the following being easy to learn: qualitative assessment of LV function (i.e. > or < EF 40%) 37, 38, hydronephrosis 39, DVT 40, finding fluid 30, and assessment for B-lines or A-lines 30. In contrast, there is data to support that biliary and gallbladder pathology may be more difficult to learn 23, 41. In our study, this is less relevant as there was a very low prevalence of biliary ultrasound; however, we have applied these results to any complex hepatobiliary application such as evaluation for cirrhosis or hepatomegaly, as these often require more technique and skill. In a Malawian study, DVT exams were considered “easy”; FASH, heart, and renal exams were considered “moderate”; and liver and gynecology exams were considered “difficult” 2. In that study, the FASH exam is considered moderate difficulty likely due to the inclusion of the assessment of splenic abscesses and abdominal lymphadenopathy. In a separate paper, the study authors outlined the “FASH-basic” which focuses only on finding fluid in the pleural and abdominal spaces and likely requires significantly less skill 22. Thus, the following applications have met the criteria of “easy to learn”: 1) FASH-basic or finding fluid, both of which include pericardial effusion, pleural effusion, ascites/abdominal free fluid, 2) qualitative assessment of LV function, and 3) A-lines vs. B-lines. Contrarily, we determined that FASH-plus, assessment for consolidation, and evidence of cirrhosis would be more challenging to perform, and thus we recommend excluding these applications from the initial curriculum (Figure 4).

### Impact on Diagnosis and Management 

Of the remaining indications (Figure 4), finding fluid changed the diagnosis and management 63.4% and 65.9% of the time, respectively, and qualitative assessment of LV function did so 54.2% and 52.1% of the time, respectively. Contrarily, the FASH exam changed the diagnosis and management 22% and 24%, respectively, and A-lines/B-lines/Consolidation did so 26% and 18.5% of the time, respectively. Thus, the following applications have been defined as higher impact: qualitative assessment of LV function and finding fluid. The following have been excluded as lower impact: FASH basic and A-lines vs. B-lines. All clinical questions and their respective impact can be seen in Table 3. 

### Quality Assurance and Validation of Data

**Figure 5:**
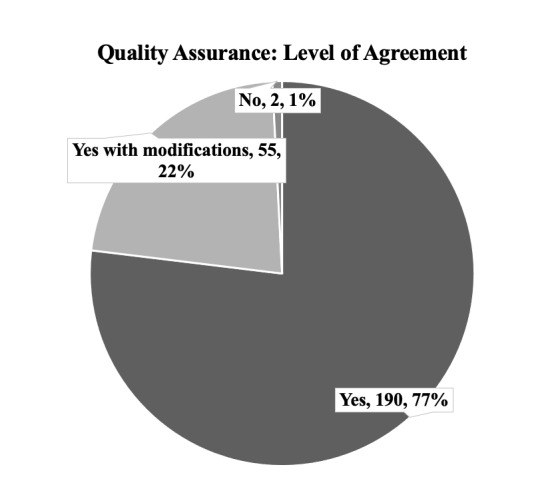
Level of agreement between the reviewer and the resident interpretation based on subjective report by individuals reviewing the scans.

A total of 243 (94.9%) of scans were reviewed by experts for quality assurance. Of those that were reviewed, 76.9% had complete agreement between the resident and reviewer and 22.3% noted agreement but with modifications (Figure 5). In 2 (0.8%) cases the reviewer did not agree with the interpretation, though this did not change the clinical management in either case. Of note, reviewer agreement with the interpretation did not significantly differ between clinical questions. The 13 scans that were not reviewed either had local quality assurance by a radiologist or were not uploaded correctly to the Google Drive and were thus unable to be reviewed. More detailed analysis of the quality assurance of these scans can be seen in Fox et al 29.

## Discussion

In our study, POCUS was able to answer the clinical question 92% of the time and changed diagnosis and management 41.8% and 41.4% of the time, respectively. This is comparable to other studies that have been done in LMICs 23, 32, 33, 34, 42. Questions that were more difficult to answer with POCUS were more open-ended, such as “etiology of abdominal pain” and “evaluation for malignancy.” Binary questions such as “is there evidence of a pleural effusion?” were more likely to answer the question. This is consistent with prior literature discussing the most effective use of POCUS 2, 33, 43, 44.

### Proposed Indications to Include in GH POCUS Curriculum

Based on the results above (Figure 4), we have outlined a recommended curriculum for POCUS education of IM practitioners in LMICs settings similar to those in this study (Table 4). This includes assessment for free fluid and qualitative assessment of LV function. One important note is that the FASH exam is most sensitive when utilized in patients with HIV and CD4 counts less than 100. It is possible that in our study the FASH exam was performed in a broader population, which may have decreased its sensitivity and specificity 22. Thus, in settings where there is a high prevalence of HIV-TB coinfection, we recommend including the FASH-basic exam into the curriculum as well, which would mainly consist of teaching how finding fluid can be applied to the diagnosis of TB in patients with HIV, particularly those with CD4 counts less than 100. In such settings, changing the diagnosis and management even 15-20% of the time would arguably be worthwhile. 

**Table 4:** Proposed Basic Curriculum for Internal Medicine practitioners in LMICs.

**POCUS Application**	**Clinical questions**	**Scanning locations**
**Finding fluid** *Including FASH-basic exam in areas of high HIV/TB prevalence	Is there a pericardial effusion? Is there evidence of a pleural effusion? Is there evidence of ascites or abdominal free fluid?	Subxyphoid view Bilateral lung bases Right upper quadrant Left upper quadrant Suprapubic
**LV function**	What is the qualitative left ventricular function?	Parasternal long axis Apical 4 chamber view Subxyphoid view

For assessment of LV function, we recommend emphasizing that the goal of this assessment is to evaluate general, or qualitative, heart function rather than measuring ejection fraction or assessing more complex valvular pathology. We recommend still obtaining a formal echocardiogram in most cases with the knowledge that this may take several days to get done in these settings, or patients may not be able to be transported for it at all. Depending on skill level, assessment of LV function may be incorporated with the assessment of B-lines and pleural effusions to form the Cardiac Limited Ultrasound (CLUE) exam^45^ to determine overall volume responsiveness or need for diuresis, though this may be too nuanced for basic learners.

Two applications that we did not include in our proposed curriculum but may be useful are assessment of hydronephrosis and assessment for DVT. We did not include these because the prevalence in our study sites was quite low, but in areas where the prevalence is higher, these applications may be worthwhile to include and would meet the ease of learning criteria. One additional application that was found to be useful in Malawi was the assessment of kidney size. Often, it is difficult to obtain lab results in a timely manner, so kidney size and character was often used as a surrogate marker for possible chronic kidney disease. 

### Role of Quality Assurance

It is worth briefly discussing the role of QA both for our study and for future potential curricula. For our study, QA served two purposes: 1) to validate the results of our study, and 2) to increase the quality of our residents’ education while abroad, as described in our other paper 29. Ideally, QA would be incorporated into any POCUS curriculum, but we recognize this may not be possible in many centers. Whenever possible, learners should be encouraged to review their deidentified images with a more expert individual, whether that be in person or electronically via mobile applications.

### Limitations

Our study has several limitations. First, our study discusses POCUS applications in LMICs; however, there is of course substantial heterogeneity of clinical setting within LMICs, including different disease prevalence/epidemiology and resource availability. It should be noted that a significant majority of scans in our study were performed in Malawi over Guyana, likely due to a decreased number of rotators in Guyana as well as more formal imaging resources available in Guyana. As such, it is worth emphasizing that the study may have limited generalizability to all LMICs. 

Second, the data collected was subjective report. While we attempted to standardize this by providing criteria for diagnosis and management change, there is still potential for variation in what constitutes “changed diagnosis” and “changed management” per participant. 

Third, we did not pre-define clinical questions that were appropriate for POCUS, which resulted in some open-ended questions, such as “etiology of abdominal pain” and “evaluation for malignancy” that were more difficult to answer with POCUS. In the future, we would standardize these to include more specific, binary questions. We suspect this may be due to the fact that in settings with limited availability of alternative imaging, POCUS frequently is used to answer more broad questions rather than the binary clinical questions that are answered in high-resource settings. 

Finally, while we validated our findings with QA, we did not measure patient outcomes in our study, nor did we measure the feasibility of this curriculum being implemented among local practitioners. Next steps for this project would be to teach and include local practitioners, measuring the feasibility and applicability not only with US-trained IM residents but with local IM practitioners, allowing for capacity building and sustained integration of POCUS, which would be the gold standard for assessing whether the diagnostic and management change was valid. 

### Conclusions and Next Steps

In this study, we recommend that an initial POCUS curriculum for inpatient medicine practitioners in LMIC settings similar to those in this study include the following applications: finding fluid (pericardial effusion, pleural effusion, and ascites) and qualitative assessment of LV function. This novel educational model describes POCUS applications that are highest yield to include in an IM POCUS curriculum based on prevalence, impact, and ease of use, and could improve the way POCUS is taught and used in these settings. 

## Disclosures

The authors declare no conflicts of interest. 
